# Chinas Greater Bay Area

**DOI:** 10.1007/s10273-021-2901-x

**Published:** 2021-04-17

**Authors:** Britta Kuhn

**Affiliations:** Wiesbaden Business School der Hochschule RheinMain, Bleichstr. 44, 65183 Wiesbaden, Deutschland

**Keywords:** F15, F21, O53

## Abstract

Die Volksrepublik China will wichtige Städte ihrer südöstlichen Küstenprovinz Guangdong eng mit Hongkong und Macao verbinden. Geplant ist ein integrierter Wirtschaftsraum, der bis spätestens 2035 eine globale Führungsrolle einnehmen soll. Die Grundidee ist nicht neu, nimmt aber zunehmend Gestalt an. Es gibt allerdings auch große Hindernisse.

## Abstract (en)

Economic cooperation among mainland China, Hong Kong and Macao has deepened quickly since 2019. A roadmap for China’s southeastern province Guangdong promotes a common development of nine municipalities within the Pearl River Delta and the two Special Administrative Regions. The Chinese government wants this combined Greater Bay Area to become a highly innovative city cluster with world-class technologies and industries. Fully integrated transport networks and many pilot schemes are on their way. They will probably end the “one country, two systems“-principle much before schedule. However, the ambitious plan faces impediments such as political tensions, trade confl icts and the COVID-19 pandemic.

Im Februar 2019 wurde in Beijing der „Outline Development Plan for the Guangdong-Hong Kong-Macao Greater Bay Area" verkündet. Er soll die beiden Sonderverwaltungszonen Hongkong und Macao eng mit neun Städten des Perlflussdeltas vernetzen (Plan, 2019). Zur Greater Bay Area (GBA) gehören als zwei weitere „Kernstädte" Guangzhou und Shenzhen; daneben Foshan, Dongguan, Zhongshan und Zhuhai im Zentrum, Huizhou im Osten sowie Zhaoqing und Jiangmen im Westen (vgl. Abbildung 1). Die gesamte Agglomeration erwirtschaftete schon 2019 mit mehr als 70 Mio. Bewohnern ein BIP von 1,7 Billionen US-$ (vgl. Tabelle 1). Zum Vergleich: Der Großraum Tokio kam auf knapp 2 Billionen US-$ (2017), die San Francisco Bay Area auf 0,9 Billionen US-$ (2018) (HKTDC Research).

### Von Deng Xiaoping zum Entwicklungsplan

Maos Nachfolger Deng errichtete Chinas erste Sonderwirtschaftszonen Ende der 1970er Jahre ganz bewusst in der Nähe von Hongkong, Macao und Taiwan. Ab den 1990er Jahren prägten Ökonom:innen dann Begriffe wie „Greater Hong Kong" oder „South China Economic Zone". 2003 entstanden die ersten Freihandelsabkommen zwischen Hongkong bzw. Macao und der Volksrepublik. Diese „Closer Economic Partnership Arrangements" (CEPA) öffneten Unternehmen der beiden Sonderverwaltungszonen den Marktzutritt zum gesamten chinesischen Festland in ausgewählten Produktions- und Dienstleistungssektoren. Praxisrelevant waren die Abkommen vor allem für das benachbarte Perlflussdelta. 2008 veröffentlichte Chinas Entwicklungs- und Reformkommission (NDRC) einen Entwicklungsplan für diesen festlandchinesischen Teil der heutigen GBA. 2014 verabschiedete Chinas Staatsrat die „China (Guangdong) Pilot Free Trade Zone" (GDFTZ). Die GDFTZ experimentierte mit weiteren Handelsliberalisierungen zwischen Guangdong, Hongkong und Macao. 2017 folgte von der NDRC und den beteiligten Lokalverwaltungen ein Rahmenabkommen über eine vertiefte Kooperation zwischen Guangdong, Hongkong und Macao. Im Februar 2019 konkretisierte wiederum Chinas Staatsrat die Entwicklungsstrategie mit dem bislang einschlägigen Entwicklungsplan (2022 Foundation, 2019; Hirn, 2020).

### Elf Partnerstädte

Tabelle 1 zeigt, wie unterschiedlich bevölkerungsreich und wirtschaftsstark die Kooperationspartner sind. Weitere erhebliche Unterschiede liegen in ihrer bisherigen Branchenstruktur und künftigen strategischen Bedeutung innerhalb der Entwicklungsplanung.[1] Hongkongs Stellung als internationales Finanz-, Transport- und Handelszentrum soll wachsen – z. B. im Flugverkehr, bei Logistik-Dienstleistungen und im Geschäft mit Offshore Renminbi. Anders als in Guangdong existiert in der ehemaligen britischen Kolonie freier Kapitalverkehr und der Hongkong-Dollar ist vollkommen konvertibel. Alle großen Banken befinden sich daher traditionell vor Ort, was die Volksrepublik seit Jahren für schrittweise Kapitalmarktöffnungen nutzt. Beispielsweise können internationale Investierende schon länger über Hongkonger Banken chinesische Aktien und Anleihen kaufen (Rohde, 2020b). Macao, bisher vor allem ein Spielerparadies, soll sich zu einem Tourismus- und Freizeitzentrum von Weltklasse entfalten, als Handelsplattform mit China und portugiesisch sprechenden Ländern dienen und seine Wirtschaftsstruktur diversifizieren.

1 Die folgenden Städteprofile beruhen, soweit nicht anders angegeben, auf Plan (2019) und Hirn (2020).

**Prof. Dr. Britta Kuhn** lehrt Volkswirtschaftslehre an der Wiesbaden Business School der Hochschule RheinMain.

Abbildung 1
**Teilnehmerstädte der Greater Bay Area**

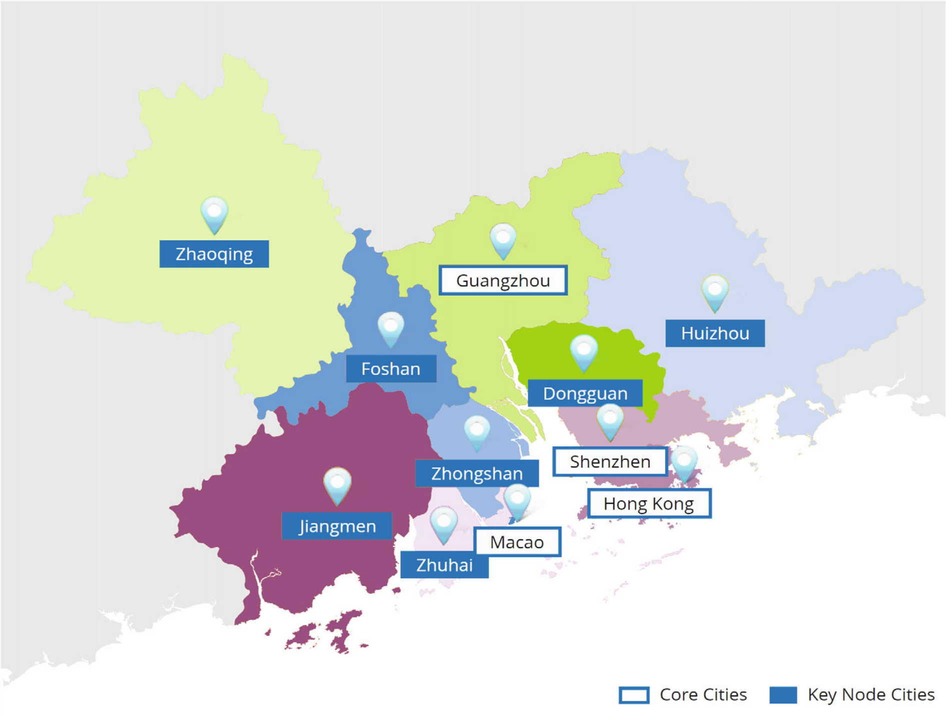

Quelle: Constitutional and Mainland Affairs Bureau.

Shenzhen gehört zu den vier Sonderwirtschaftszonen, die Deng Xiaoping 1979 schuf. Hongkongs Nachbar verfügt über eine überaus innovative Start-up-Szene. Privatunternehmen prägen die Forschungslandschaft, nicht etwa Hochschulen und Staatsfirmen. In Shenzhens Stadtteilen Luohu, Futian und Nanshan entstanden zukunftsweisende Branchencluster. Weltbekannte Firmen wie Huawei, Tencent und Ping An haben in Shenzhen ihren Hauptsitz. Die Metropole ist auch dafür bekannt, dass dort fast alle Taxis und Busse elektrisch fahren. Sie soll eine der innovativsten und kreativsten Städte von globaler Bedeutung werden.

Guangzhou gehört neben Beijing, Shanghai und Shenzhen zu den „First-Tier-Cities“ der Volksrepublik, ist also schon heute besonders groß und reich. Guangdongs Provinzhauptstadt verfügt bereits über eine Mischung aus Großunternehmen und Start-ups. Das politische Machtzentrum der GBA entwickelt sich dynamisch und wird zu einem internationalen Handels- und Dienstleistungszentrum ausgebaut.

Zhuhai befand sich wie Shenzhen unter den ersten Sonderwirtschaftszonen. Dennoch war Macaos Nachbar bisher relativ bevölkerungsarm und verfügte kaum über Unternehmen von Weltrang. Die Stadt zeichnet sich allerdings durch viele Parks, Strände und eine allgemein hohe Lebensqualität aus. Außerdem gehört Hengqin dazu. Diese Insel bietet freies Internet und soll viele Themenparks erhalten. Daneben wird sie zum Hightech-Standort für Weltkonzerne wie Tencent, Alibaba und Huawei entwickelt, etwa durch ein Forschungszentrum für Künstliche Intelligenz. Aber auch der Elektronikkonzern Foxconn plant hier für 9 Mrd. US-$ eine Mikrochip-Fertigung.

Foshan ist eine traditionelle Industriestadt mit zahlreichen Spezialclustern. Hier entstehen z. B. weiße Ware, Aluminium oder Möbel in großer Zahl. Midea fertigt ebenso in Foshan wie Volkswagen. Guangzhous kleiner Nachbar will unter anderem vom Boom bei Elektroautos profitieren. Pilotprojekte dienen dazu, den Industriestandort zu transformieren und dadurch aufzuwerten. Das gilt auch für Dongguan, wo traditionelle Massenfertigung durch Highendwaren und -dienstleistungen ersetzt werden soll – inklusive Forschung und Entwicklung in neuen Branchen. Huawei betreibt hier bereits ein Forschungszentrum.

Zhongshan war trotz seiner zentralen Lage bisher ein eher durchschnittlicher Produktionsstandort. Aber auch Chinas Lampenzentrum soll innovative Technologien herstellen, z. B. in der Biomedizin. Im Gegensatz dazu lag Huizhou lange abseits, nämlich jenseits der Verbindung von

Tabelle 1
**Kennzahlen der Greater Bay Area Städte, 2019**

| | BIP in Mrd. US-$ (US-$ pro Kopf) | Bevölkerung in Mio. | Nennungen im Entwicklungsplan |
|---|---|---|---|
| GBA | 1.679* (23.116) | 72,7 | |
| Hongkong | 366 (48.713) | 7,5 | 508 |
| Macao | 54 (79.977) | 0,7 | 539 |
| Shenzhen | 390 (29.498) | 13,4 | 110 |
| Guangzhou | 343 (22.676) | 15,3 | 82 |
| Zhuhai | 50 (25.400) | 2,0 | 42 |
| Foshan | 156 (19.102) | 8,2 | 17 |
| Dongguan | 137 (16.309) | 8,5 | 13 |
| Zhongshan | 45 (13.439) | 3,4 | 19 |
| Huizhou | 61 (12.473) | 4,9 | 11 |
| Jiangmen | 46 (9.885) | 4,6 | 13 |
| Zhaoqing | 33 (7.819) | 4,2 | 9 |

Anmerkungen: *Summe der ungerundeten Einzelwerte.

Quellen: eigene Darstellung; HKTDC Research; Plan, 2019

Shenzhen über Dongguan nach Guangzhou. Inzwischen geht es von Huizhou im Hochgeschwindigkeitszug binnen 30 Minuten nach Shenzhen und in einer Stunde nach Hongkong. Wesentliche Entwicklungsziele stellen Tourismus und grüne Landwirtschaft dar.

Jiangmen und Zhaoqing schließlich repräsentieren das Hinterland der GBA. Beide verfügen noch über viel unbebautes Land, niedrige Immobilienpreise und moderate Löhne. Besondere Förderung erhält Jiangmens Küstenbereich Yinhu Bay: Hier sollen sich internationale Konzerne, Energie- und Umweltunternehmen ebenso ansiedeln wie Start-ups und Innovationszentren. Damit will Chinas Regierung weitere Investitionen anziehen, aber auch talentierte Chines:innen aus Hongkong, Macao und Übersee. Generell soll eine Art kulturelles Austausch- und Kooperationszentrum Auslandschinesen über Jiangmen zurück in die Volksrepublik holen.

## Übergeordnete Entwicklungsziele

Bis 2022 will China die jeweiligen Stärken der GBA zusammenführen, die Branchenstrukturen optimieren und so ein hochinnovatives Städte-Cluster von Weltgeltung formen. Bis 2035 sollen Innovationen den wesentlichen Entwicklungsmotor darstellen, sodass die ökonomischen und technologischen Stärken der Region ihre Wettbewerbsfähigkeit und ihren Einfluss international weiter stärken. Der Plan sieht deshalb unter anderem vor, die regionale Entwicklung zu koordinieren und die Märkte für Güter und Produktionsfaktoren miteinander zu verbinden. Die enge Zusammenarbeit soll die Einwohner:innen der GBA wohlhabender machen, Chinas kulturellen Einfluss nachweisbar stärken und ein international erstklassiges Umfeld zum Arbeiten, Leben und Reisen schaffen.

Hongkong, Macao, Guangzhou und Shenzhen bilden das Herz des Entwicklungsplans. Die anderen sieben Partnerstädte werden mit diesen Zentren eng verwoben – vor allem mit den beiden Sonderverwaltungszonen. Beispielsweise sollen die Reisezeiten zwischen den Hauptstädten der GBA auf eine Stunde oder weniger sinken. Dafür baut die chinesische Regierung vor allem das Schienen- und Straßennetz zwischen den Partnern weiter aus und schafft zusätzliche Hochgeschwindigkeitsverbindungen (Plan, 2019). Schon 2018 lag die durchschnittliche Reisezeit innerhalb der GBA knapp unter einer Stunde. Fast 80 % der Ziele ließen sich in weniger als 90 Minuten erreichen. In die westliche Peripherie dauerte es aber im Extremfall fast fünf Stunden (Cao et al., 2019). Seither ist viel passiert. So weihte Xi Jinping Ende 2018 eine sechsspurige Straßenverbindung ein, die den direkten Weg zwischen Zhuhai, Macao und Hongkong überbrückt bzw. untertunnelt. Seit Ende 2019 geht es außerdem im Hochgeschwindigkeitszug von Zhuhais Insel Hengqin in 20 Minuten nach Shenzhen. Ebenfalls seit 2019 verbindet eine achtspurige Brücke Dongguan mit Guangzhous expandierendem Stadtteil Nansha – nicht zu verwechseln mit Nanshan in Shenzhen. 2024 soll eine ebenfalls achtspurige Brücken-Tunnel-Konstruktion die Fahrzeit zwischen Shenzhen und Zhongshan verkürzen. Daneben plant China viele weitere Bahnstrecken. Schon jetzt dauert die Reise von Foshan nach Hongkong weniger als 90 Minuten (Hirn, 2020). Vom internationalen Flughafen in Hongkong nach Zhuhai soll es statt in vier Stunden in nur noch rund 45 Minuten gehen und ab 2024 von Shenzhen nach Zhongshan statt in zwei Stunden in 20 Minuten (Constitutional and Mainland Affairs Bureau, o.D.a).

Auch die Flughäfen der GBA expandieren. Hongkong International Airport wird ebenso erneuert und erweitert wie die Flughäfen in Macao, Guangzhou, Shenzhen und weiteren Städten. Guangzhou soll außerdem einen zweiten Flughafen erhalten (Plan, 2019). Bisher gibt es in der GBA sieben Flughäfen – neben den genannten noch in Zhuhai, Foshan und Huizhou. 18,5 Mrd. US-$ sollen bis 2024 allein in die Hongkonger Erweiterung fließen. Dieser Flughafen ist im interkontinentalen Personen- und Frachtverkehr schon heute sehr wichtig für die GBA. Weitere rund 8 Mrd. US-$ will die Volksrepublik in den bestehenden Flughafen Guangzhou investieren, 1,8 Mrd. US-$ in Shenzhen Airport. Allein dort wurden 2019 schon 53 Mio. Passagiere abgefertigt, 2030 sind 80 Mio. geplant (Rohde, 2020c).

Letztlich geht es Chinas Planenden darum, industrielle Stärken systematisch auf- und auszubauen, indem sie komplementäre Branchen- und Entwicklungscluster fördern. Vielfältige Kooperations- und Experimentierzonen sollen die elf Partnerstädte verbinden, wobei meistens Aktivitäten aus Hongkong und Macao auf Guangdong ausgedehnt werden – nicht umgekehrt. Die chinesische Regierung öffnet also ihre Märkte und sorgt dafür, dass die beiden Sonderverwaltungszonen wirtschaftlich in der Volksrepublik aufgehen. Beispielsweise entstehen Experimentierzonen in Shenzhens Distrikt Qianhai, Guangzhous Nansha und Zhuhais Hengqin: Junge Menschen und Mikrounternehmer:innen aus Hongkong und Macao erhalten hier Subventionen und weitere Unterstützung, um für Start-ups und Beschäftigung zu sorgen. Ausgewählte Finanzdienstleistungen der beiden Sonderverwaltungszonen werden ebenfalls gefördert, wenn sie in die drei Entwicklungszentren expandieren. So könnte Qianhai zusammen mit Hongkong und dem restlichen Shenzhen zum größten Finanzzentrum der Welt aufsteigen, während sich Macao – vor allem mit dem benachbarten Zhuhai – zum integrierten chinesischen Freizeit-, Tourismus- und Innovationszentrum ausbilden würde. Hierzu dienen unter anderem die Projekte „Macao-Hengqin Youth Entrepreneurship Valley", der „Guangdong-Macao Cooperation Industrial Park" auf Hengqin und ein „Traditional Chinese Medicine Science and Technology Industrial Park" in Zhuhai. Macao soll aber auch intensiv mit Zhongshan zusammenarbeiten sowie mit Hongkong in Jiangmens „Greater Guanghai Bay economic zone" in Bereichen wie Finanzen, Tourismus, Kultur, E-Commerce, Gesundheit und Berufsbildung kooperieren. Schließlich ist beabsichtigt, die Seeverbindung zwischen Huizhou, Shenzhen, Hongkong und Macao touristisch zu erschließen.

Im Vergleich zu den Gemeinschaftsprojekten mit Hongkong bzw. Macao nehmen sich die konkreten Pläne innerhalb Guangdongs bescheiden aus: Westlich des Perlflusses soll z. B. ein fortschrittlicher Industriegürtel mit Zhuhai und Foshan als Speerspitzen entstehen. Am Ostufer lautet das Ziel, Shenzhen, Dongguan und Umgebung zum Fertigungscluster von weltweiter Bedeutung auszubauen – unter anderem für hochwertige Elektronikgüter (Plan, 2019).

## Mögliche Entwicklungshindernisse

Bislang gelten in Hongkong, Macao und im Perlflussdelta drei unterschiedliche Rechts- und Verwaltungssysteme. Der GBA fehlt insofern auch eine gemeinsame Verwaltungsebene – elf Städte müssen sich miteinander abstimmen (Rohde, 2020b). Jenseits des geopolitischen Themas „Rechtsstaatlichkeit und Demokratie" verfügen die beiden Sonderverwaltungszonen bisher insbesondere über eigene Steuer- und Zollbestimmungen, eigene Vorschriften zu Visum und Arbeitserlaubnis sowie über eigene Währungen (Hirn, 2020). Auf dem Weg zu einem integrierten GBA-Binnenmarkt müssten also noch zahlreiche Hürden im Waren-, Dienstleistungs-, Kapital-, Daten- und Personenverkehr abgebaut werden. Ökonom:innen aus Hongkong, Guangdong und Europa haben daher vorgeschlagen, sich an der Europäischen Union zu orientieren: Die jeweiligen Vorschriften könnten erstens über Mindeststandards, gegenseitige Anerkennung und Harmonisierung den Grundsatz „ein Land, zwei Systeme" untermauern. Der EU-Ansatz sei flexibel und minimiere dadurch mögliche Spannungen zwischen den Partnern. Die Wirtschaftsexpert:innen empfehlen zweitens, wie die EU über wissenschaftliche, technische, Umwelt-, Sozial- und viele weitere Gemeinschaftsprogramme zu kooperieren. Auch dies schränke die jeweilige Souveränität nicht ein (2022 Foundation, 2019).

Tatsächlich birgt der politische Grundsatz „ein Land, zwei Systeme" allerdings viel Sprengstoff. Zwar zitiert die chinesische Regierung dieses Prinzip, das Deng Xiaoping prägte, oft in ihrem Entwicklungsplan. Dessen Präambel erklärt aber auch, dass die vollständige Integration Hongkongs und Macaos in das chinesische Festland das übergeordnete Ziel darstellt. Der Plan (2019) solle den „Kompatrioten" der beiden Sonderverwaltungszonen unter anderem dabei helfen, langfristig reich und stabil zu bleiben und mit der dortigen Bevölkerung „den Stolz eines starken und florierenden Mutterlandes" zu teilen. Beijing versteht das Prinzip „ein Land, zwei Systeme" also bestenfalls rein ökonomisch, solange die Vorteile greifbar sind. Z. B. verabschiedete Beijing fast gleichzeitig zum umstrittenen Sicherheitsgesetz für Hongkong ein Pilotprojekt, das den freien Kapitalverkehr innerhalb der GBA bald verbessern soll. Im Gegensatz zu bisherigen Programmen umfasst es also nicht die gesamte Volksrepublik, sondern nur das Perlflussdelta, Hongkong und Macao. Es wird grenzüberschreitende Investitionen in Vermögensverwaltungsprodukte ermöglichen. Dies dürfte vor allem den Finanzplatz Hongkong begünstigen. Er leidet zunehmend unter Vertrauensproblemen, wovon wiederum Singapur profitiert (Rohde, 2020b). Sollte das Hongkonger Entwicklungsmodell weiter verblassen, dürfte auch der wirtschaftliche Sonderstatus der ehemaligen Kronkolonie zügig erodieren. Der Konvergenzprozess – nach EU-Vorbild? – wäre dann nicht erst 2047 abgeschlossen, wenn Hongkong seinen Selbstverwaltungsstatus aus vertraglichen Gründen allerspätestens verliert.

Drohen in Macao ähnliche Unruhen und Repressionen wie in Hongkong, bevor auch hier der 1999 vereinbarte Sonderstatus nach 50 Jahren ohnehin enden wird? Eher nicht: Sympathisanten der Kommunistischen Partei Chinas kontrollieren die ehemalige portugiesische Kolonie schon seit mehr als 50 Jahren politisch und wirtschaftlich. Ihre lokale Bevölkerung erlebte zu keinem Zeitpunkt einen starken Rechtsstaat, demokratische Wahlrechte oder eine unabhängige Medienberichterstattung. Macaos Einwohner:innen identifizieren sich aber wohl auch deshalb viel stärker mit der Volksrepublik, weil das dortige BIP pro Kopf seit der Unabhängigkeit in 1999 wesentlich stärker wuchs als in Hongkong (Des Garets Geddes, 2020).

Schließlich könnten Handelskonflikte und die Corona-Pandemie die ehrgeizigen Pläne für die GBA ausbremsen. Der Optimismus hat jedenfalls in den vergangenen zwei bis drei Jahren erheblich gelitten: Erstens divergierte die wirtschaftliche Entwicklung zwischen den Sonderverwaltungszonen und dem benachbarten Festlandchina. Während Hongkong und Macao schrumpften, ging es im Perlflussdelta deutlich bergauf. Zweitens verlagerten Hongkonger Unternehmen Fabriken vom chinesischen Festland nach Südostasien, um dem Handelsstreit zwischen den USA und China zu entkommen. Drittens führte die Corona-Pandemie zu vollständigen Grenzschließungen und einer starken Diversifizierung – von chinesischen Produzenten zu alternativen Lieferländern (Rohde, 2020a). Auch jenseits von Handelskonflikten und Pandemie dürfte die Diversifizierung globaler Lieferketten noch jahrelang weitergehen. Speziell in Macao brach das BIP ein, weil Tourismus und Spielbankgeschäft zum Erliegen kamen (Rohde, 2020b). Insgesamt ist die GBA also ein ehrgeiziges Projekt, das den wirtschaftlichen Sonderstatus Hongkongs und Macaos lange vor 2047 bzw. 2049 begrenzen dürfte, aber vermutlich weniger erfolgreich verlaufen wird als von Beijing geplant.

**Title:** ***China's Greater Bay Area***

**JEL Classification:** *F15, F21, O53*
